# Expression of Heparan Sulfate Endosulfatases in the Adult Mouse Brain: Co-expression of *Sulf1* and Dopamine D1/D2 Receptors

**DOI:** 10.3389/fnana.2021.726718

**Published:** 2021-08-20

**Authors:** Ken Miya, Kazuko Keino-Masu, Takuya Okada, Kenta Kobayashi, Masayuki Masu

**Affiliations:** ^1^Graduate School of Comprehensive Human Sciences, University of Tsukuba, Tsukuba, Japan; ^2^Department of Molecular Neurobiology, Division of Biomedical Science, Faculty of Medicine, University of Tsukuba, Tsukuba, Japan; ^3^Section of Viral Vector Development, Center for Genetic Analysis of Behavior, National Institute for Physiological Sciences, National Institutes of Natural Sciences, Okazaki, Japan

**Keywords:** sulfatase 1, sulfatase 2, expression, *in situ* hybridization, mouse brain, dopamine receptor, *Sulf1*

## Abstract

The heparan sulfate 6-*O*-endosulfatases, Sulfatase 1 (Sulf1), and Sulfatase 2 (Sulf2), are extracellular enzymes that regulate cellular signaling by removing 6-*O*-sulfate from the heparan sulfate chain. Although previous studies have revealed that *Sulfs* are essential for normal development, their functions in the adult brain remain largely unknown. To gain insight into their neural functions, we used *in situ* hybridization to systematically examine *Sulf1/2* mRNA expression in the adult mouse brain. *Sulf1* and *Sulf2* mRNAs showed distinct expression patterns, which is in contrast to their overlapping expression in the embryonic brain. In addition, we found that *Sulf1* was distinctly expressed in the nucleus accumbens shell, the posterior tail of the striatum, layer 6 of the cerebral cortex, and the paraventricular nucleus of the thalamus, all of which are target areas of dopaminergic projections. Using double-labeling techniques, we showed that *Sulf1*-expressing cells in the above regions coincided with cells expressing the dopamine D1 and/or D2 receptor. These findings implicate possible roles of *Sulf1* in modulation of dopaminergic transmission and dopamine-mediated behaviors.

## Introduction

Heparan sulfate proteoglycans (HSPGs) are glycoproteins present on the cell surface and in the extracellular matrix (ECM) of all animal cells. In the nervous system, they play critical roles in neuron growth, differentiation, migration, axon guidance, synapse formation, and synaptic plasticity (Holt and Dickson, 2005; Condomitti and de Wit, 2018; Kamimura and Maeda, 2021). HSPGs are composed of a core protein and covalently attached heparan sulfate (HS) chains. HSPGs exert a wide variety of biological functions via the interactions among HS and growth factors, morphogens, ECM molecules, and enzymes (Perrimon and Bernfield, 2000; Bishop et al., 2007). During their biosynthesis, HS chains undergo a series of modifications including deacetylation, epimerization, and sulfation, which together generate the enormous structural heterogeneity of HS (Perrimon and Bernfield, 2000; Bishop et al., 2007). In addition, further processing can occur by the HS 6-*O*-endosulfatases, Sulfatase 1 (Sulf1), and Sulfatase 2 (Sulf2). Sulfs remove 6-*O*-sulfate from HS extracellularly, thereby regulating various cellular signaling pathways positively or negatively (Morimoto-Tomita et al., 2002; Lamanna et al., 2007; Vivès et al., 2014; El Masri et al., 2017). For example, cell signaling by heparin-binding growth factors, such as fibroblast growth factor and vascular endothelial growth factor, is attenuated by Sulf-mediated HS desulfation (Wang et al., 2004; Narita et al., 2006). Conversely, Sulfs promote canonical Wnt signaling by releasing Wnt ligands as a result of the decrease in Wnt–HS interaction (Dhoot et al., 2001; Ai et al., 2003). Thus, Sulfs are considered to be essential for fine-tuning of HS functions under normal physiological conditions and their dysfunction leads to pathological consequences such as carcinogenesis and developmental abnormalities.

Accumulating evidence from animal studies has revealed the functional significance of *Sulf* genes *in vivo*. *Sulf1/2* knockout mice showed skeletal, renal, lung, inner ear, and neuronal defects during development (Holst et al., 2007; Lum et al., 2007; Ratzka et al., 2008; Freeman et al., 2015). In the development of the nervous system, *Sulfs* are implicated in specification of oligodendrocyte precursors (Danesin et al., 2006; Touahri et al., 2012; Jiang et al., 2017), esophageal innervation (Ai et al., 2007), neurite outgrowth and migration of cerebellar neurons (Kalus et al., 2015), and axon guidance in the corticospinal tract (Okada et al., 2017). Although the roles of *Sulfs* in development have been extensively studied, their significance in adult brain functions remains largely unknown. Given that HSPGs are key components of synapse-organizing protein complexes and the well-known presynaptic organizers Neurexins are synthesized as HSPGs, it is possible that Sulfs are involved in synapse assembly, maturation, and plasticity through HS modification (Condomitti and de Wit, 2018; Zhang et al., 2018; Kamimura and Maeda, 2021). Previously, we reported that in the adult rat brain, *Sulf1* mRNA is expressed in the cerebral cortex, olfactory tubercle, hypothalamus, and choroid plexus (Ohto et al., 2002), and *Sulf2* mRNA, in the cerebral cortex, hippocampus CA3 region, and medial habenula (Nagamine et al., 2005). However, their expression patterns throughout the entire brain and the types of cells expressing *Sulfs* have not been studied in detail. Therefore, to gain insight into their neural functions, we used *in situ* hybridization to examine their mRNA expression patterns in the adult mouse brain. We found that *Sulf1* and *Sulf2* exhibit spatially distinct expression patterns. In addition, we found that *Sulf1*-expressing cells coincided with the cells expressing the dopamine D1/D2 receptors. These data will provide a clue for elucidating the roles of *Sulf* genes in higher brain functions.

## Materials and Methods

### Mice

*Sulf1* and *Sulf2* knockout (KO) mice were generated as previously described (Nagamine et al., 2012). Both *Sulf1* and *Sulf2* genes were independently disrupted by inserting a cassette of stop-IRES-lacZ-polyA into their exons using homologous recombination in ES cells. The offspring of mice backcrossed to C57BL/6N for 10 and 14 successive generations for *Sulf1* and *Sulf2*, respectively, were used. Two transgenic lines, Tg(Drd1-cre)EY262Gsat/Mmucd (Drd1-Cre) and Tg(Drd2-cre)ER44Gsat/Mmucd (Drd2-Cre), were obtained from the Mutant Mouse Resource and Research Centers (MMRRC). These mice, originally generated in the FVB/N strain (Gong et al., 2003; Heintz, 2004), were maintained in the C57BL/6J background in the MMRRC and further backcrossed to C57BL/6N for 10 successive generations in our laboratory. Two other yellow-fluorescent-protein (YFP) transgenic lines (Nagai et al., 2016), C57BL/6J-Tg(mDrd1-YFP)680-1Koba (Drd1-YFP) and B6.Cg-Tg(Drd2-YFP)364-5 (Drd2-YFP), were obtained from the RIKEN BioResource Research Center (Tsukuba, Ibaraki, Japan). Genotypes were determined by means of PCR using genomic DNA isolated from mouse tails. All animal experiments were approved by and performed according to the guidelines of the Animal Care and Use Committee of the University of Tsukuba.

### *In situ* Hybridization

Adult male mice (8–12 weeks old, total seven mice) were transcardially perfused with 4% paraformaldehyde (PFA)/phosphate-buffered saline (PBS) while under deep anesthesia induced by intraperitoneal injection of overdose of pentobarbital sodium. The brains were extracted and postfixed with 4% PFA/PBS at 4°C overnight. After cryoprotection with 30% sucrose in PBS, the brains were embedded in Tissue-Tek OCT compound (Sakura Finetek Japan, Tokyo, Japan), frozen, and stored at –25°C. The brains were cut with a cryostat (CM 1850; Leica Biosystems, Wetzlar, Germany) into 50-μm coronal or sagittal slices, collected in 2-ml tubes filled with a cryoprotectant solution (30% glycerol, 30% ethylene glycol, 40% PBS), and stored at –25°C until use. Immediately before hybridization, the slices were washed with PBS with 0.1% Tween-20 (PBST), treated with Proteinase K (1 μg/ml) at 37°C for 5 min, washed with PBST for 1 min three times, and fixed in 4% PFA/PBST for 20 min. After being washed with PBST for 1 min three times, the slices were hybridized with a digoxigenin (DIG)-labeled RNA probe (1 μg/ml) in hybridization solution (50% formamide, 5 × saline-sodium citrate buffer [SSC] pH 4.5, 1% sodium dodecyl sulfate [SDS], 50 μg/ml heparin, 50 μg/ml yeast RNA) at 65°C overnight. After being washed with 50% formamide, 5 × SSC pH 4.5, 1% SDS at 65°C for 30 min; with 50% formamide, 2 × SSC pH 4.5 at 65°C for 30 min three times; and with Tris-buffered saline with 0.1% Tween-20 (TBST) for 5 min three times, the slices were incubated with TBST containing 0.5% blocking reagent (Roche Diagnostics, Mannheim, Germany) for 60 min at room temperature, and then with alkaline phosphatase-conjugated anti-DIG antibody (1:2000; Roche Diagnostics) in TBST containing 0.5% blocking reagent at 4°C overnight. After being washed with TBST for 20 min three times, and then with 100 mM NaCl, 50 mM MgCl_2_, 100 mM Tris-HCl pH 9.5, 0.1% Tween-20 for 5 min, signals were detected with BM purple (Roche Diagnostics) in the presence of 2 mM levamisole (Sigma-Aldrich, St. Louis, MO, United States) at room temperature for 24 h. The probe contained the sequence 2,810–3,730 for *Sulf1* (NM_001198565.1) and the sequence 1,743–2,390 for *Sulf2* (NM_028072.5). The slices were washed and mounted on MAS-coated slide glasses (Matsunami Glass Industry, Osaka, Japan), and the coverslips were mounted using Fluoromount-G (Southern Biotech, Birmingham, AL, United States). Images were recorded using a digital microscope (Biozero BZ-8000; Keyence, Osaka, Japan) and a microscope (Axioplan 2; Carl Zeiss, Jena, Germany). *Sulf1* or *Sulf2* KO mice were used as negative controls. Brain regions were identified by reference to *The Mouse Brain in Stereotaxic Coordinates* by Franklin and Paxinos (2008) and to the *Allen Brain Atlas*^1^.

### Stereotactic Surgery

Adeno-associated virus (AAV) vectors were packaged using the AAV Helper Free Expression System (Cell Biolabs, San Diego, CA, United States). Briefly, the packaging plasmids (pAAV-RC5 and pHelper) and pAAV-hSyn-DIO-mCherry were transfected into HEK293T cells by means of the calcium phosphate method (Sano et al., 2020). A crude lysate was purified by means of serial ultracentrifugation with cesium chloride. The purified virus particles were dialyzed and concentrated with an Amicon 10K MWCO filter (Merck Millipore, Darmstadt, Germany). The copy number of the viral genome was determined by means of real-time PCR (approximately 1.1 × 10^13^ viral genome/ml).

Adult male mice (9–38 weeks old, total 10 mice) were used for viral injection. The mice were anesthetized with a mixture of midazolam, medetomidine, and butorphanol (4, 0.75, and 5 mg/kg body weight) and head-fixed on a stereotaxic frame (David Kopf Instruments, Tujunga, CA, United States). After craniotomy, an AAV vector was injected using Micro-Hematocrit Capillary Tubes (Fisher Scientific, Pittsburgh, PA, United States) and a pressure microinjector (KDS 101; Muromachi, Tokyo, Japan) at a rate of 100 nl/min. The stereotactic coordinates and injection volumes were as follows: for the nucleus accumbens shell (NAcSh), anterior-posterior (AP) 1.6 mm, medial-lateral (ML) 0.6 mm, dorsal-ventral (DV) 4.0 mm, 1 μl; for the posterior tail of the striatum (TS), AP −1.0 mm, ML 3.2 mm, DV 3.2 mm, 500 nl; for the prefrontal cortex (PFC), AP 2.0 mm, ML 0.4 mm, DV 2.4 mm, 500 nl; and for the paraventricular nucleus of the thalamus (PVT), AP−1.2 mm, ML 0.45 mm, DV 3.2 mm with a 10° angle toward the midline, 500 nl. The injection needle was withdrawn 5 min after the end of the injection. The mice were fixed by perfusion 2 weeks after the injection.

### Immunohistochemistry

Adult male mice (8–40 weeks old, total 21 mice) were transcardially perfused with 4% PFA/PBS while under deep anesthesia induced by intraperitoneal injection of overdose of pentobarbital sodium. The extracted brains were postfixed with 4% PFA/PBS at 4°C overnight. The brains were cut into 50-μm coronal slices by use of a vibratome (VT1000 S; Leica Biosystems). After the slices were serially dehydrated through 25–80% methanol/PBST and then serially rehydrated into PBST, they were incubated with primary antibodies in PBST containing 0.5% blocking reagent (Roche Diagnostics) at room temperature overnight. The slices were washed with PBST for 15 min three times, and then incubated with secondary antibodies in PBST containing 0.5% blocking reagent at room temperature for 2 h. The primary antibodies used were anti-green fluorescent protein (GFP) (A11122, 1:1000 dilution; Molecular Probes, Eugene, OR, United States), anti-β-galactosidase (4600-1409, 1:1000 dilution; Biogenesis, Poole, United Kingdom), anti-NeuN (MAB377, 1:500 dilution; Chemicon International, Temecula, CA, United States), and anti-S100 beta (ab41548, 1:1000 dilution; Abcam, Cambridge, United Kingdom). The secondary antibodies used were Alexa Fluor Plus 488- or Alexa Fluor Plus 594-conjugated donkey anti-goat IgG (A32814/A32758, 1:500 dilution; Invitrogen, Rockford, IL, United States), Alexa Fluor 488-conjugated donkey anti-rabbit IgG (A21206, 1:500 dilution; Molecular Probes), Cy3-conjugated donkey anti-mouse IgG (715-165-150, 1:200 dilution; Jackson ImmunoResearch, West Grove, PA, United States), and Cy3-conjugated donkey anti-rabbit IgG (AP182C, 1:200 dilution; Chemicon International). Nissl staining was done by incubation of the slices with NeuroTrace 530/615 Red Fluorescent Nissl Stain (Molecular Probes) diluted at 1:200 in PBST for 20 min after anti-β-galactosidase staining.

### Image Acquisition and Analysis

The fluorescently labeled brain slices were observed and photographed using laser scanning confocal microscopy (LSM 700; Carl Zeiss, Jena, Germany). Low magnification images were acquired using a 10x objective lens. For each region of interest (ROI), a series of z-stack fluorescence images (1-μm intervals) were recorded using a 20x objective lens. Cells positive for β-galactosidase and GFP (or mCherry) in each ROI were counted. Briefly, Imaris software (Bitplane, Zurich, Switzerland) was used to mark cells positive for β-galactosidase with spheres in 1 layer and cells positive for GFP (or mCherry) with squares in a different layer. After independent marking, the two layers were overlaid, and cells that were doubly or singly marked with spheres and squares were counted (see Supplementary Figure 3).

## Results

### Specificity of the Probes

To examine the expression of *Sulf1* and *Sulf2* mRNA in the adult mouse brain, *in situ* hybridization was performed using digoxigenin-labeled antisense RNA probes specific to each sequence and a chromogenic substrate, BM purple. As summarized in Table 1, the *Sulf1* signals were abundant in the neocortex, limbic cortex, basal ganglia, and some thalamic and hypothalamic nuclei, whereas the *Sulf2* signals were seen in the neocortex, limbic cortex, septum, habenula, hypothalamus, and lower brainstem. Largely, if not entirely, *Sulf1* and *Sulf2* showed distinct expression patterns (Supplementary Figure 1), indicating no cross hybridization. More importantly, the *Sulf1* and *Sulf2* signals were completely abolished in the *Sulf1* KO and *Sulf2* KO mice, respectively (Supplementary Figure 1), indicating the specificity of the probes. The expression patterns of *Sulf1* and *Sulf2* are described in greater detail in the following sections.

**TABLE 1:** Expression of Sulf1 and Sulf2 in the adult mouse brain.

Brain area	*Sulf1*	*Sulf2*	Brain area	*Sulf1*	*Sulf2*
Olfactory system			Thalamus		
Olfactory bulb			Anterodorsal nucleus	+	–
Granule cell layer	+	–	Paratenial nucleus	+	–
Mitral cell layer	–	++	Paraventricular nucleus		
Outer plexiform layer	+ (sparse)	–	Anterior part	+++	+
Glomerular layer	+ (sparse)	++	Posterior part	+++	–
Anterior olfactory nucleus	+++	+++	Interanterodorsal nucleus	++	–
Olfactory tubercle	+++	–	Intermediodorsal nucleus	+++	–
Neocortex			Central medial nucleus	+++	–
Layer 5	–	+++	Paracentral nucleus	+	–
Layer 6a	– ∼ +	+	Rhomboid nucleus	+	–
Layer 6b	+ ∼ +++	++	Nucleus of reuniens	++	–
Limbic cortex			Xiphoid nucleus	+++	–
Piriform cortex	+++	–	Parafascicular nucleus	+++	–
Tenea tecta			Reticular nucleus	–	+
Dorsal part	++	++	Peripeduncular nucleus	+	–
Ventral part	+	–	Hypothalamus		
Entorhinal cortex	+++	+++	Anteroventral periventricular nucleus	+	+
Subiculum			Median preoptic nucleus	++	+
Dorsal part, pyramidal cell	–	+++	Suprachiasmatic nucleus	–	+
Ventral part, pyramidal cell	+	+++	Zona incerta	–	+
Dentate gyrus			Paraventricular nucleus	++	+
Polymorph layer	–	++	Dorsomedial nucleus	+++	+
Hippocampus			Lateral hypothalamic area	+	+
CA1, pyramidal cell	–	+	Posterior hypothalamic nucleus	+	–
CA3, pyramidal cell	–	+++	Arcuate nucleus	+++	+
Septum			Parasubthalamic nucleus	+	–
Lateral septal nucleus	+	+++	Mammillary nucleus	–	+
Medial septal nucleus	–	+++	Tuberomammillary nucleus, ventral part	+	–
Triangular nucleus of septum	+ (sparse)	–	Brainstem		
Dorsal peduncular cortex	–	+++	Substantia nigra	–	++ (sparse)
Bed nucleus of the stria terminalis	+ (sparse)	+ (sparse)	Ventral tegmental area	+ (sparse)	–
Diagonal band nucleus	–	+++	Interpeduncular nucleus	–	++
Magnocellular nucleus	–	+++	Darkschewitsch nucleus	–	+
Ventral pallidum	–	+	Periaqueductal gray	+ (sparse)	+
Basal ganglia			Raphe nuclei	+	+
Dorsal striatum			Anterior tegmental nucleus	–	+
Subventricular zone	+	–	Ventral tegmental nucleus	–	+
Posterior tail	++	–	Parabrachial nucleus	+	++
Nucleus accumbens			Locus ceruleus	–	++
Shell	+++	–	External cuneate nucleus	–	++
Claustrum	+++	++	Tegmental reticular nucleus	–	++
Endopiriform nucleus	+++	++	Pontine gray	–	++
Amygdala			Motor nucleus of trigeminal	–	+
Basomedial amygdalar nucleus	+	–	Cochlear nuclei	–	++
Basolateral amygdalar nucleus	–	+	Inferior olivary complex	–	++
Habenula			Nucleus of the solitary tract	+	++
Medial habenula	–	+++	Lateral reticular nucleus	–	+
Circumventricular organ			Cerebellum		
Vascular organ of the lamina terminalis	+	+	Deep cerebellar nuclei	+ (sparse)	+++
Subfornical organ	++	+	Purkinje cell layer	++	–
Median eminence	++	–	Choroid plexus	+++	–
Area postrema	+++	++			

*Distributions of Sulf1 and Sulf2 mRNAs in the adult mouse brain are shown. No or very weak expression is labeled as (−), weak expression as (+), moderate expression as (++), and strong expression as (+++).*

### *Sulf1* Expression in the Olfactory System and Striatum

**FIGURE 1:**
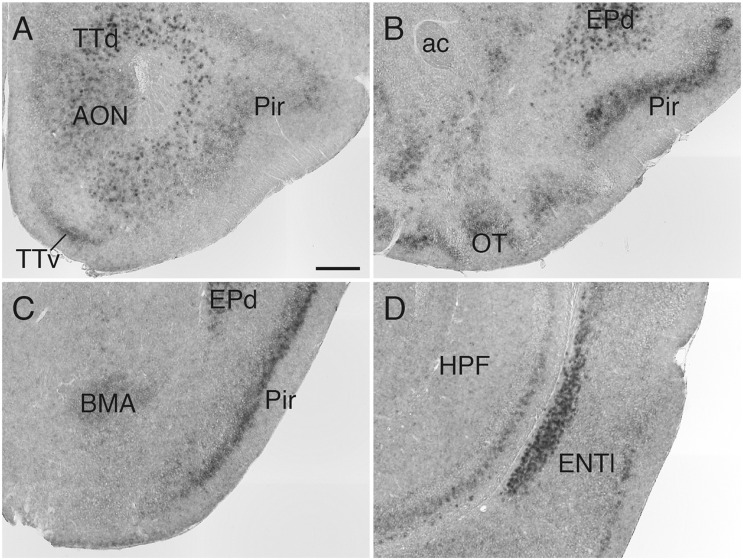
*Sulf1* mRNA expression in the olfactory cortex. **(A–D)** *Sulf1* was expressed in the anterior olfactory nucleus (AON), tenia tecta (TTd, TTv), olfactory tubercle (OT), piriform cortex (Pir), dorsal endopiriform nucleus (EPd), and lateral entorhinal cortex (ENTl). ac, anterior commissure; BMA, basomedial amygdalar nucleus; HPF, hippocampal formation. The scale bar indicates 250 μm. Approximate AP levels from the bregma (in mm) are 2.5 **(A)**, 1.0 **(B)**, –1.5 **(C)**, and −3.0 **(D)**.

In the olfactory system, *Sulf1* mRNA was detected in the anterior olfactory nucleus, tenia tecta, olfactory tubercle, piriform cortex, and lateral entorhinal cortex (Figure 1). *Sulf1* expression was observed through the entire length of the olfactory tubercle and piriform cortex along the rostrocaudal axis.

**FIGURE 2:**
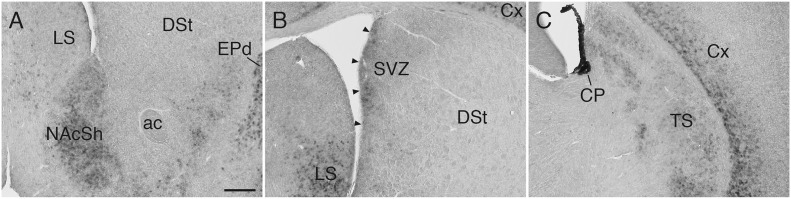
*Sulf1* mRNA expression in the striatum. **(A)** *Sulf1* was expressed in the nucleus accumbens shell (NAcSh). **(B)** *Sulf1* was detected in the subventricular zone (SVZ) of the lateral ventricle (arrowheads) and the ventral part of the lateral septal nucleus (LS). **(C)** *Sulf1* was observed in the posterior tail of the striatum (TS) and layer 6 of the cerebral cortex (Cx). The strongest *Sulf1* expression was observed in the choroid plexus (CP). ac, anterior commissure; DSt, dorsal striatum; EPd, endopiriform nucleus dorsal part. The scale bar indicates 250 μm. Approximate AP levels from the bregma (in mm) are 1.0 **(A)**, 0 **(B)**, and –1.0 **(C)**.

In the striatum, *Sulf1* was highly expressed in the shell, but not core, of the nucleus accumbens (NAc): *Sulf1* expression was high in the medial shell and low in the lateral shell (Figure 2A). *Sulf1* was undetectable in the dorsal striatum except for in the following regions: the subventricular zone facing the lateral ventricle (Figure 2B) and the most caudal part of the dorsal striatum, which corresponds to the posterior tail of the striatum (TS; Figure 2C).

### *Sulf1* Expression in the Cerebral Cortex and Subcortical Regions of the Forebrain

**FIGURE 3:**
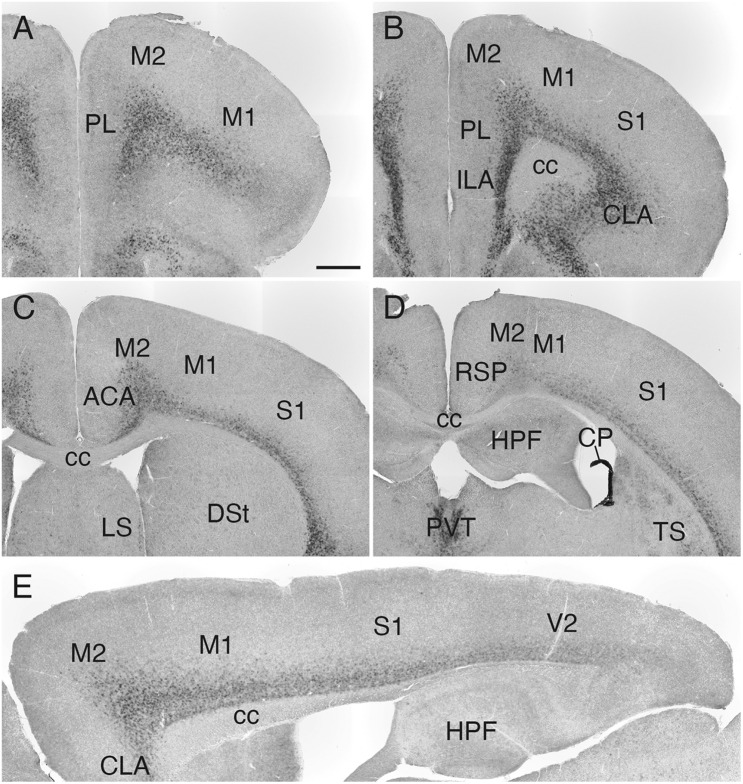
*Sulf1* mRNA expression in the cerebral cortex. **(A–D)** *Sulf1* was expressed in layer 6 of the cerebral cortex. Coronal sections from the rostral **(A)** to the caudal **(D)** levels are shown. **(E)** Sagittal section showing the *Sulf1* expression in the cerebral cortex throughout the rostrocaudal axis. The expression showed a high-rostral to low-caudal gradient. ACA, anterior cingulate area; cc, corpus callosum; CLA, claustrum; CP, choroid plexus; DSt, dorsal striatum; HPF, hippocampal formation; ILA, infralimbic area; LS, lateral septal nucleus; M1, primary motor area; M2, secondary motor area; PL, prelimbic area; PVT, paraventricular nucleus of the thalamus; RSP, retrosplenial area; S1, primary somatosensory area; TS, posterior tail of the striatum; V2, secondary visual cortex. The scale bars indicate 500 μm **(A–D)** and 400 μm **(E)**. Approximate AP levels from the bregma (in mm) are 2.5 **(A)**, 2.0 **(B)**, 0.5 **(C)**, and –1.0 **(D)**. The approximate ML level from the midline (in mm) is 1.0 **(E)**.

**FIGURE 4:**
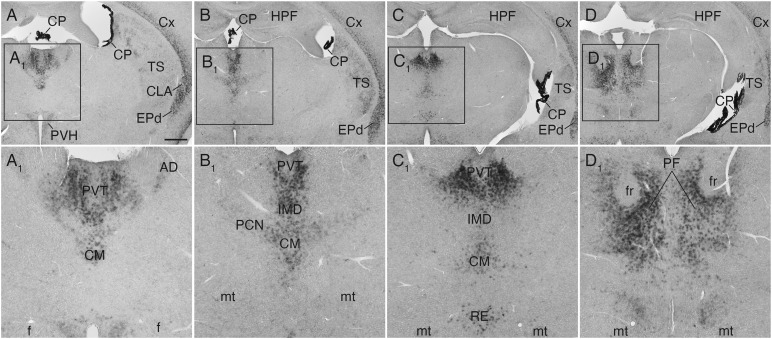
*Sulf1* mRNA expression in the thalamus. **(A–D)** *Sulf1* was detected in the midline thalamic nuclei, including the paraventricular (PVT), intermediodorsal (IMD), and reuniens (RE) nuclei. It was also seen in the intralaminar nuclei, including the central medial (CM), paracentral (PCN), and parafascicular (PF) nuclei. **(A_1_−D_1_)** Show the enlarged images in the boxed areas in **(A–D)**, respectively. AD, anterodorsal nucleus; CLA, claustrum; CP, choroid plexus; Cx, cerebral cortex; EPd, endopiriform nucleus dorsal part; f, fornix; fr, fasciculus retroflexus; HPF, hippocampal formation; mt, mammillothalamic tract; PVH, paraventricular hypothalamic nucleus; TS, posterior tail of the striatum. The scale bars indicate 500 μm **(A–D)** and 200 μm **(A_1_–D_1_)**. Approximate AP levels from the bregma (in mm) are –0.5 **(A)**, –1.0 **(B)**, –2.0 **(C)**, and –2.5 **(D)**.

*Sulf1* mRNA was detected throughout the cerebral cortex (Figures 3A–D), and its expression had a high-rostral to low-caudal gradient (Figure 3E and Supplementary Figure 2). More specifically, in the cerebral cortex, the most prominent expression was observed in the prefrontal cortex (PFC), and weaker expression was seen in the caudal cortex (Figures 3A–D and Supplementary Figure 2). In all of these areas, the expression was confined to layers 6: strong in layer 6b and weaker in layer 6a (Figures 2–4 and Supplementary Figure 2).

In the thalamus, *Sulf1* was highly and selectively expressed in the midline group including the paraventricular, intermediodorsal, and reuniens nuclei (Figures 4A–C,A_1_–C_1_), and in the intralaminar group including the central medial, paracentral, and parafascicular nuclei (Figures 4A–D,A_1_–D_1_). *Sulf1* expression was observed from the anterior parts to the posterior parts of the paraventricular nucleus of the thalamus (PVT; Figures 4A–C,A_1_–C_1_). *Sulf1* was also strongly expressed in the claustrum and dorsal endopiriform nucleus (Figures 1B,C, 2A, 3B,E, 4A–D,A_1_–D_1_). The strongest *Sulf1* expression in the brain was observed in the choroid plexus (Figures 2–4).

### *Sulf1* Expression in the Septum and Hypothalamus

**FIGURE 5:**
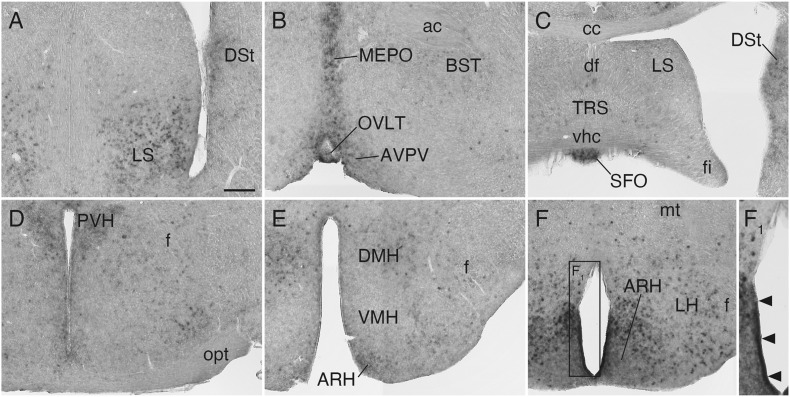
*Sulf1* mRNA expression in the septum and hypothalamus. **(A,B)** *Sulf1* was expressed in the ventral part of the lateral septal nucleus (LS), median preoptic nucleus (MEPO), and organum vasculosum of the lamina terminalis (OVLT). **(C)** *Sulf1* was detected in the subfornical organ (SFO). **(D–F)** In the hypothalamus, *Sulf1* was expressed in the paraventricular (PVH), dorsomedial (DMH), and arcuate (ARH) nuclei and in the lateral hypothalamic area (LH). **(F_1_)** Shows the enlarged image in the boxed area in **(F)**. The arrowheads in **(F_1_)** indicate strong *Sulf1* expression in the cells facing the ventral portion of the third ventricle. ac, anterior commissure; AVPV, anteroventral periventricular nucleus; BST, bed nucleus of the stria terminalis; cc, corpus callosum; df, dorsal fornix; DSt, dorsal striatum; f, fornix; fi, fimbria; mt, mammillothalamic tract; opt, optic tract; TRS, triangular nucleus of septum; vhc, ventral hippocampal commissure; VMH, ventromedial nucleus of the hypothalamus. The scale bars indicate 200 μm **(A–F)** and 120 μm **(F_1_)**. Approximate AP levels from the bregma (in mm) are 0.5 **(A,B)**, –0.5 **(C)**, –1.0 **(D)**, –2.0 **(E)**, and –2.5 **(F)**.

In the septal area, *Sulf1* was expressed in the ventral part of the lateral septal nucleus (Figure 5A). In the hypothalamus, moderate expression was found in the median preoptic and paraventricular nuclei (Figures 5B,D), and strong expression, in the dorsomedial and arcuate nuclei (Figures 5E,F). Furthermore, very strong *Sulf1* expression was observed in the single-layered cells facing the ventral portion of the third ventricle (Figures 5F,F_1_). On the basis of the location, this signal seems to reflect the expression in tanycytes, which are highly specialized ependymal cells (Prevot et al., 2018).

### *Sulf1* Expression in the Midbrain, Cerebellum, and Lower Brainstem

**FIGURE 6:**
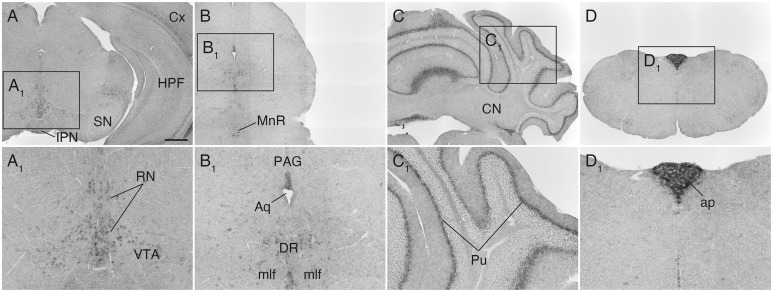
*Sulf1* mRNA expression in the midbrain, cerebellum, and lower brainstem. **(A,B)** *Sulf1* was expressed in the raphe nuclei (RN) in the midbrain, including the dorsal (DR) and median raphe (MnR) nuclei, and part of the ventral tegmental area (VTA). **(C)** In the cerebellum, *Sulf1* expression was seen in the Purkinje cell layer (Pu). **(D)** The area postrema (ap) was strongly labeled. **(A_1_–D_1_)** Show the enlarged images in the boxed areas in **(A–D)**, respectively. Aq, aqueduct; CN, cerebellar nuclei; Cx, cerebral cortex; HPF, hippocampal formation; IPN, interpeduncular nucleus; mlf, medial longitudinal fasciculus; PAG, periaqueductal gray; SN, substantia nigra. The scale bars indicate 500 μm **(A–D)** and 200 μm **(A_1_–D_1_)**. Approximate AP levels from the bregma (in mm) are –3.5 **(A)**, –4.5 **(B)**, –6.5 **(C)**, and –7.5 **(D)**.

In the lower brainstem, *Sulf1* expression was found sparsely in the ventral tegmental area (VTA), midbrain raphe nuclei, and periaqueductal gray (Figures 6A,A_1_,B,B_1_). In the cerebellum, *Sulf1* mRNA was detected in the Purkinje cell layer throughout the cortex (Figures 6C,C_1_). It should also be noted that *Sulf1* was observed in the circumventricular organs including the organum vasculosum of the lamina terminalis (Figure 5B), the subfornical organ (Figure 5C), and the area postrema (Figures 6D,D_1_).

### *Sulf2* Expression in the Adult Mouse Brain

**FIGURE 7:**
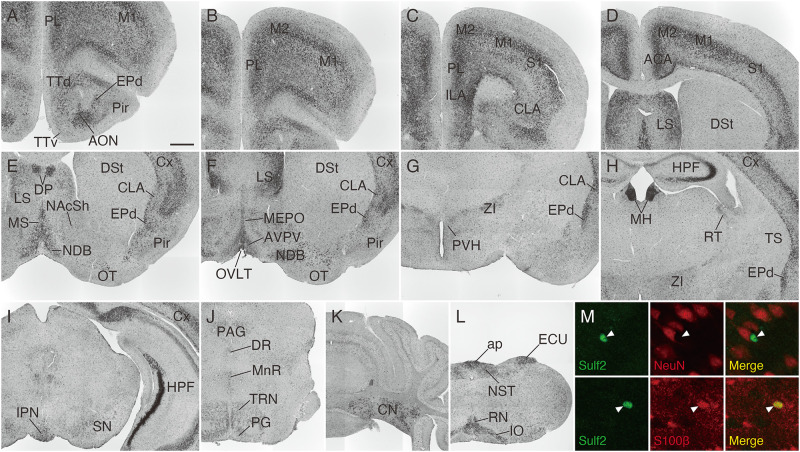
*Sulf2* mRNA expression in the adult mouse brain. **(A–L)** *Sulf2* was broadly expressed in the brain. Strong signals were observed in the cerebral cortex (Cx), lateral septal nucleus (LS), medial habenula (MH), hippocampal formation (HPF), cerebellar nuclei (CN), inferior olivary complex (IO), external cuneate nucleus (ECU), and area postrema (ap). **(M)** Confocal microscopic images of the brain slices from *Sulf2*^LacZ/+^ mice stained with anti-β-galactosidase (green) and anti-NeuN or S100β (red). Representative images of the scattered punctate signals in the thalamic region are shown. The β-galactosidase signals, indicative of *Sulf2* expression, were co-localized with an astrocyte marker, S100β (arrowheads, lower panels), but not with a neuronal marker, NeuN (arrowheads, upper panels). ACA, anterior cingulate area; AON, anterior olfactory nucleus; AVPV, anteroventral periventricular nucleus; CLA, claustrum; DP, dorsal peduncular cortex; DR, dorsal raphe nucleus; DSt, dorsal striatum; EPd, endopiriform nucleus dorsal part; ILA, infralimbic area; IPN, interpeduncular nucleus; M1, primary motor area; M2, secondary motor area; MEPO, median preoptic nucleus; MnR, median raphe nucleus; MS, medial septal nucleus; NAcSh, nucleus accumbens shell; NDB, diagonal band nucleus; NST, nucleus of the solitary tract; OT, olfactory tubercle; OVLT, organum vasculosum of the lamina terminalis; PAG, periaqueductal gray; PG, pontine gray; Pir, piriform cortex; PL, prelimbic area; PVH, paraventricular hypothalamic nucleus; RN, raphe nuclei; RT, reticular nucleus of the thalamus; SN, substantia nigra; S1, primary somatosensory area; TRN, tegmental reticular nucleus; TS, posterior tail of the striatum; TTd, tenia tecta dorsal part; TTv, tenia tecta ventral part; ZI, zona incerta. The scale bars indicate 500 μm **(A–L)** and 25 μm **(M)**. Approximate AP levels from the bregma (in mm) are 2.5 **(A,B)**, 2.0 **(C)**, 0.5 **(D,F)**, 1.0 **(E)**, –1.0 **(G)**, –1.5 **(H,M)**, –3.5 **(I)**, –4.5 **(J)**, –6.5 **(K)**, and –7.5 **(L)**.

We then analyzed *Sulf2* mRNA expression in the adult mouse brain. As shown in Figure 7, *Sulf2* was detected throughout the brain and its expression was high in the cerebral cortex, lateral septal nucleus, medial habenula, hippocampal CA3 region, cerebellar nuclei, area postrema, external cuneate nucleus, and inferior olivary complex. In the cerebral cortex, *Sulf2* was expressed strongly in layer 5 and layer 6b, and weakly in layer 6a (Supplementary Figures 2A,B), whereas *Sulf1* expression was restricted to layer 6. In addition to neuronal expression, punctate signals were scattered throughout the brain regions. These signals overlapped with an astrocyte marker, S100β, but not with a neuronal marker, NeuN (Figure 7M), suggesting *Sulf2* expression in astrocytes. These data show that *Sulf1* and *Sulf2* expressions differed considerably, although they were overlapped in some regions (Table 1).

### Co-localization of Sulf1 and Dopamine Receptors

Our results demonstrated an interesting correlation between *Sulf1* mRNA expression and the brain regions that receive dopaminergic signals. We therefore focused on identifying the co-localization of Sulf1 and dopamine receptors. For this purpose, we used two different strategies to examine at the cellular level whether *Sulf1*-expressing cells matched up with cells expressing the dopamine D1 receptor (D1R) or the D2 receptor (D2R).

**FIGURE 8:**
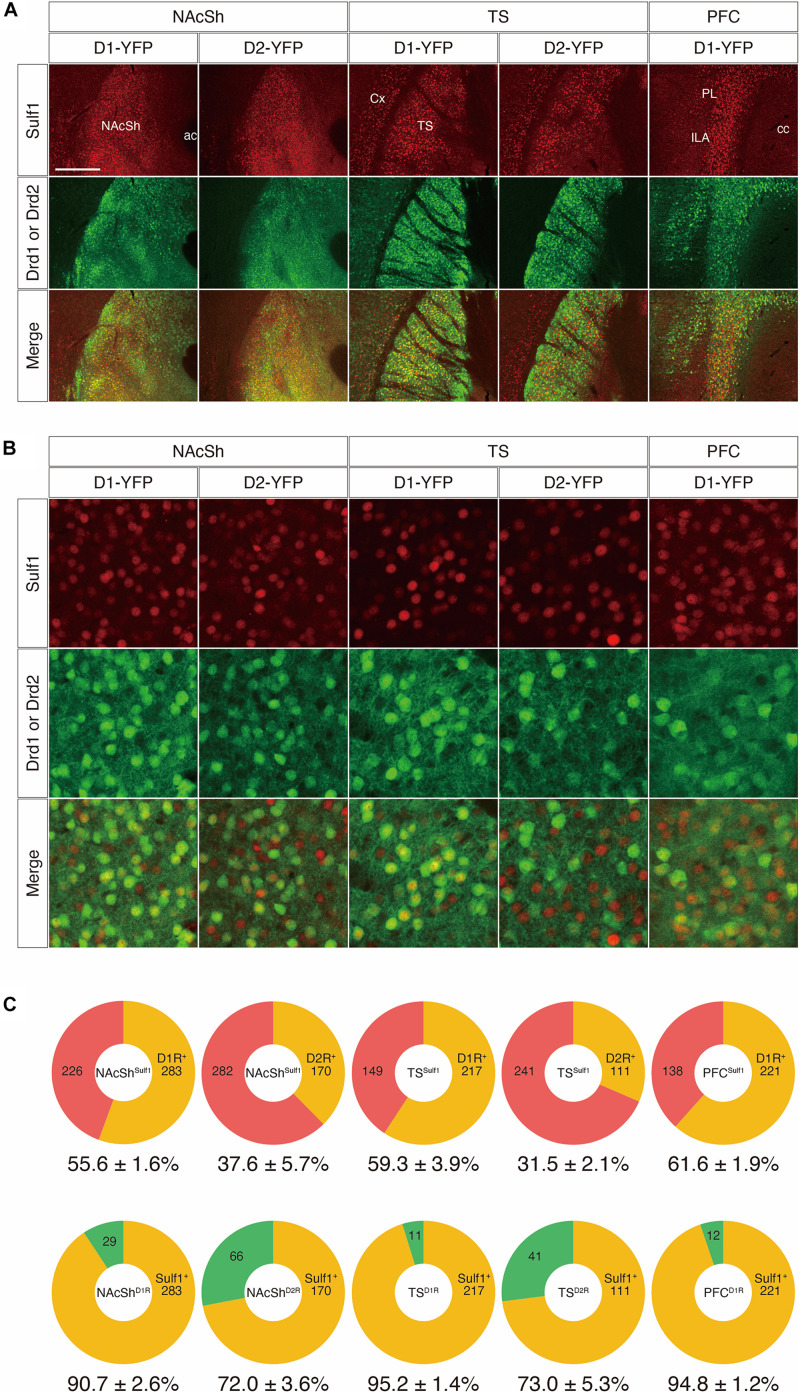
Co-expression of Sulf1 and the dopamine D1/D2 receptors. **(A)** Confocal microscopic images of the coronal brain slices. Representative images from the nucleus accumbens shell (NAcSh), posterior tail of the striatum (TS), and prefrontal cortex (PFC) are shown. *Sulf1*-expressing cells were identified by immunostaining β-galactosidase (red) by means of lacZ inserted in the *Sulf1* locus (*Sulf1*^lacZ/+^). D1R- or D2R-expressing neurons were identified by immunostaining GFP (green) in Drd1- or Drd2-YFP mice. Co-localization of Sulf1 and Drd1/2 were clearly observed. ac, anterior commissure; cc, corpus callosum; Cx, cerebral cortex; ILA, infralimbic area; PL, prelimbic area. **(B)** High magnification images of the NAcSh, TS, and PFC regions after immunostaining β-galactosidase (red) and GFP (green) are shown. **(C)** The upper panels show the percentages of D1R- or D2R-positive (orange) and D1R- or D2R-negative (red) cells in the *Sulf1*-expressing cells. The lower panels show the percentages of *Sulf1*-positive (orange) and *Sulf1*-negative (green) cells in the D1R- or D2R-expressing cells. Cell counting was conducted for two randomly selected ROIs for all regions from three mice. The numbers in the circular graphs indicate the number of cells in each category. The numbers below the graphs indicate the percentages of co-localization. The scale bars indicate 400 μm **(A)** and 50 μm **(B)**. Approximate AP levels from the bregma (in mm) in **(A,B)** are 1.0 (NAcSh), –1.0 (TS), and 2.0 (PFC).

First, we examined co-localization using mouse strains that carry both *lacZ* in the *Sulf1* locus and either a Drd1-yellow-fluorescent-protein (YFP) or a Drd2-YFP transgene (Sulf1^lacZ/+^;Drd1-YFP or Sulf1^lacZ/+^;Drd2-YFP). In these mice, because an IRES-lacZ cassette was inserted into the *Sulf1* gene to disrupt it (Nagamine et al., 2012), *lacZ* expression faithfully delineates *Sulf1*-expressing cells. Moreover, as β-galactosidase has a nuclear localization signal and the protein is located in the cell nucleus, it enables us to identify the positive cells easily. The Drd1-YFP and Drd2-YFP lines possess transgenes carrying a YFP gene under the promoters of the *Drd1* and *Drd2* genes, which encode the dopamine D1R and D2R, respectively (Nagai et al., 2016). As shown in Figure 8A, the patterns of β-galactosidase immunostaining completely matched the patterns of *Sulf1* mRNA distribution revealed by *in situ* hybridization. In addition, the distribution was overlapping with YFP signals in D1R- or D2R-expressing cells. We then examined the co-localization of β-galactosidase and YFP by means of confocal microscopy (Figure 8B; see the procedure details in the section “Materials and Methods” and Supplementary Figure 3). As shown in Figure 8C, among the *Sulf1*-expressing cells in the NAcSh (NAcSh^Sulf1^), 55.6 and 37.6% were positive for YFP in the Drd1-YFP and Drd2-YFP mice, respectively. Similarly, among the *Sulf1*-expressing cells in the TS (TS^Sulf1^), 59.3 and 31.5% were positive for YFP in the Drd1-YFP and Drd2-YFP mice, respectively (Figure 8C). Finally, among the *Sulf1*-expressing cells in the PFC (PFC^Sulf1^), 61.6% were positive for YFP in the Drd1-YFP mice (Figure 8C). In an opposite manner, the ratio of the *Sulf1*-positive cells in the YFP-expressing cells in the Drd1-YFP mice was 90.7% in the NAcSh, 95.2% in the TS, and 94.8% in the PFC, whereas the ratio of the *Sulf1*-positive cells in the YFP-expressing cells in the Drd2-YFP mice was 72.0% in the NAcSh and 73.0% in the TS (Figure 8C). Co-localization of Sulf1 and D2R in the PVT could not be examined because YFP was not expressed in the PVT of the Drd2-YFP mice, although *Drd2* mRNA is expressed in the PVT (Clark et al., 2017). Therefore, we decided to use other strains, as described in the next paragraph.

**FIGURE 9:**
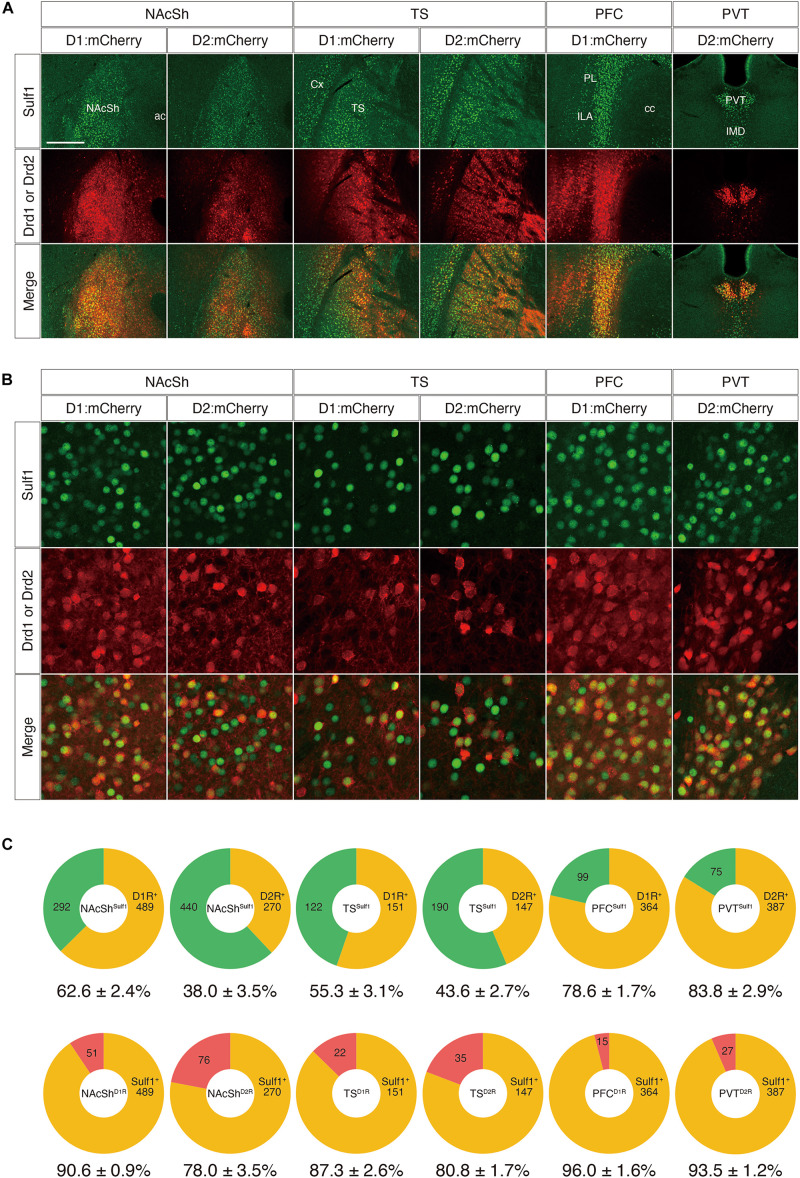
Co-expression of Sulf1 and the dopamine D1/D2 receptors. **(A)** Confocal microscopic images of the coronal brain slices. *Sulf1*-expressing cells were identified by detection of β-galactosidase inserted in the *Sulf1* locus (*Sulf1*^lacZ/+^ mice). To label the D1R- or D2R-expressing neurons, AAV5-hSyn-DIO-mCherry was injected into the specific brain regions of the mice carrying Drd1-Cre or Drd2-Cre transgenes. The signals for β-galactosidase (green) and mCherry (red) are shown. The yellow signals in the merged images indicate co-localization. Coronal slices of the nucleus accumbens shell (NAcSh), posterior tail of the striatum (TS), prefrontal cortex (PFC), and paraventricular nucleus of the thalamus (PVT) are shown. ac, anterior commissure; cc, corpus callosum; Cx, cerebral cortex; ILA, infralimbic area; IMD, intermediodorsal nucleus of the thalamus; PL, prelimbic area. **(B)** High magnification images of the NAcSh, TS, PFC, and PVT regions are shown. **(C)** The upper panels show the percentages of D1R- or D2R-positive (orange) and D1R- or D2R-negative (green) cells in the *Sulf1*-expressing cells in each region. The lower panels show the percentages of *Sulf1*-positive (orange) and *Sulf1*-negative (red) cells in the D1R- or D2R-expressing cells in each region. Cell counting was conducted for three randomly selected ROIs for all regions from three mice (NAcSh) and two mice (other regions). The numbers in the circular graphs indicate the number of cells in each category. The numbers below the graphs indicate the percentages of co-localization. The scale bars indicate 400 μm in **(A)** and 50 μm in **(B)**. Approximate AP levels from the bregma (in mm) in **(A,B)** are 1.0 (NAcSh), –1.0 (TS, PVT), and 2.0 (PFC).

Next, to examine the co-localization of Sulf1 and D2R in the PVT and to strengthen the above results, we adopted a second strategy using other mouse strains that carry both *lacZ* in the *Sulf1* locus and either a Drd1-Cre or a Drd2-Cre transgene (Sulf1^lacZ/+^;Drd1-Cre or Sulf1^lacZ/+^;Drd2-Cre). The Drd1-Cre and Drd2-Cre lines possess transgenes carrying a *Cre* gene under the promoters of the *Drd1* and *Drd2* genes, respectively. These mice are extensively used to study the anatomy and physiology of the striatal signaling pathways (Valjent et al., 2009). Injection of AAV5-hSyn-DIO-mCherry into these strains resulted in the Cre-mediated recombination of DIO (double-floxed inverted open reading frame) and led to mCherry expression in *Drd1*- and *Drd2*-expressing cells (Figure 9A). When AAV5-hSyn-DIO-mCherry virus was injected into the NAcSh, TS, PFC, and PVT, many mCherry-positive cells were observed in the corresponding regions, and the mCherry signals were overlapping with the *Sulf1* expression revealed by β-galactosidase immunostaining (Figure 9A). We then examined the co-localization with β-galactosidase and mCherry at the cellular level (Figure 9B). As shown in Figure 9C, among the *Sulf1*-expressing cells in the NAcSh (NAcSh^Sulf1^), 62.6 and 38.0% were positive for *Drd1* and *Drd2*, respectively. Similarly, among the *Sulf1*-expressing cells in the TS (TS^Sulf1^), 55.3% was positive for *Drd1*, and 43.6%, for *Drd2* (Figure 9C). Among the *Sulf1*-expressing cells in the PFC (PFC^Sulf1^), 78.6% were positive for *Drd1*, whereas among the *Sulf1*-expressing cells in the PVT (PVT^Sulf1^), 83.8% were positive for *Drd2* (Figure 9C). In an opposite manner, the ratio of the *Sulf1*-positive cells in the *Drd1*-expressing cells was 90.6% in the NAcSh (NAcSh^D1R^), 87.3% in the TS (TS^D1R^), and 96.0% in the PFC (PFC^D1R^), whereas *Sulf1*-expression in the *Drd2*-expressing cells was 78.0% in the NAcSh (NAcSh^D2R^), 80.8% in the TS (TS^D2R^), and 93.5% in the PVT (PVT^D2R^). Taken together, these data demonstrate that *Sulf1* expression coincides with *Drd1/2* expression in the NAcSh, TS, PFC, and PVT.

## Discussion

In this study, we examined *Sulf1/2* mRNA expression throughout the adult mouse brain by using *in situ* hybridization. We found that the distribution of the two mRNAs was largely segregated, although some overlapping was observed in a few brain regions (see Table 1). Previous studies have demonstrated that disruption of the *Sulf1* or *Sulf2* gene singly did not result in apparent abnormalities, whereas disruption of the two genes together led to perinatal lethality and defects in the skeletal, renal, and neural systems (Ai et al., 2007; Holst et al., 2007; Ratzka et al., 2008; Freeman et al., 2015; Okada et al., 2017). These results suggest functional redundancy of the *Sulf* genes during development owing to overlapping expression of the *Sulf1/2* genes in mouse embryos. Given that *Sulf1* and *Sulf2* show distinct expression patterns in the adult brain, it is possible that they have separate roles in different neural circuits dependent on their expressing regions. HSPGs act as synapse organizers and are implicated in synapse formation and neural plasticity (Holt and Dickson, 2005; Condomitti and de Wit, 2018; Kamimura and Maeda, 2021). More specifically, in the drosophila neuromuscular junction, RNAi-mediated knockdown of heparan sulfate 6-*O*-sulfotransferase (*Hs6st*) and *Sulf1* resulted in decrease and increase in the amplitude of the synaptic current, respectively (Dani et al., 2012; Kamimura and Maeda, 2021), indicating regulatory roles of HS 6-*O*-sulfation in synaptic function. Therefore, it would be intriguing to further explore the neural functions of *Sulf* genes individually, although some behavioral abnormalities were already reported in *Sulf1* and *Sulf2* KO mice (Kalus et al., 2009). Given that astrocyte-derived glypican 4, one of the HSPGs, is known to cluster AMPA receptors and promote the formation of active synapses (Kamimura and Maeda, 2021), Sulf2 may have some role in astrocyte-mediated synaptic plasticity.

*Sulf1* was distinctly expressed in the NAcSh, TS, claustrum, dorsal endopiriform nucleus, layer 6 of the cerebral cortex, and PVT. All of these regions are the target areas of the dopaminergic projections from the substantia nigra (SN), VTA, and others (Björklund and Dunnett, 2007; Li et al., 2014; Wong et al., 2021). In addition, we found that *Sulf1-*expressing cells were positive for D1R and/or D2R in the above regions (Figures 8, 9, data not shown), suggesting a close relationship between Sulf1 and D1R/D2R. From this viewpoint, it would be intriguing to explore the functions of *Sulf1* in relation to the dopamine system. Sulf1 may regulate cell surface localization of dopamine receptors and transporters, act as a co-receptor for dopamine and growth factors, and regulate diffusion of dopamine in the ECM. It is thus important to test the possibility that *Sulf1* gene disruption affects dopaminergic transmission. In addition, *Sulf1* expression in both layer 6 of the cerebral cortex and the claustrum is also intriguing, because a common developmental and evolutionary origin of these two structures has recently been advocated (Bruguier et al., 2020).

The striatum consists mostly of GABAergic projection neurons called medium spiny neurons (MSNs). They are divided into two distinct populations according to their output pathways and molecular markers (Björklund and Dunnett, 2007; Cox and Witten, 2019). They receive dense innervation from dopamine neurons in the SN and VTA and possess dopamine receptors. It was reported that in the dorsal striatum, 52% and 43% of the MSNs express D1R and D2R, respectively, and only 5% express both subtypes, whereas in the NAcSh, 47% and 36% of the MSNs express D1R and D2R, respectively, and 17% express both (Bertran-Gonzalez et al., 2008). We found that most of the D1R- or D2R-expressing neurons in the NAcSh and TS were positive for Sulf1; therefore, it is likely that *Sulf1* is expressed in most of the MSNs in these regions. The dopaminergic projection from the SN to the dorsal striatum is involved in motor and reinforcement-based behaviors, whereas the projection from the VTA to the NAc plays pivotal roles in reward- or aversion-related behaviors (Cox and Witten, 2019). The TS is the extreme caudal portion of the dorsal striatum and has unique anatomical connections and peculiar functions that are different from those of the other portions of the dorsal striatum (Menegas et al., 2015, Menegas et al., 2018; Cox and Witten, 2019; Valjent and Gangarossa, 2021). It is therefore possible that *Sulf1* is associated with appetitive/aversive behaviors, motivation, and integration of sensory information and may have a relation with psychiatric diseases and drug addiction. Future work concerning dopamine-related behaviors will be required to elucidate the contribution of *Sulf1* to higher brain functions.

In the thalamus, *Sulf1* was selectively expressed in the midline and intralaminar nuclei, which have a broad connection with the NAc, hippocampus, and amygdala (Van Der Werf et al., 2002; Kirouac, 2015; Vertes et al., 2015; Kirouac, 2021). Thus, Sulf1 may be associated with the functions of these groups of cells in stress, anxiety, and drug-seeking activity. In particular, the PVT is a region of interest because it has a massive efferent connection with the NAc and controls NAc activity through the activation of the dopaminergic signal from the VTA (Parsons et al., 2007). Because D2R is expressed in the PVT, *Sulf1* may affect the function of the PVT through dopaminergic modulation.

*Sulf1* was also expressed in the anterior olfactory nucleus, tenia tecta, olfactory tubercle, piriform cortex, and lateral entorhinal cortex. These are collectively called the olfactory cortex and receive inputs from the mitral/tufted cells of the olfactory bulb (Igarashi et al., 2012). Given that they are involved in processing and perception of odors, *Sulf1* may have some role in odorant signaling. Again, because D1R/D2R are abundant in the olfactory tubercle, piriform cortex, and lateral entorhinal cortex, *Sulf1* may regulate odor signal processing through dopaminergic modulation.

*Sulf1* was strongly expressed in the area postrema, organum vasculosum of the lamina terminalis, and subfornical organ. These organs are categorized as the sensory circumventricular organs (Sisó et al., 2010). They have highly permeable capillaries and lack the blood-brain barrier, thereby facilitating communication among the brain parenchyma, cerebrospinal fluid, and blood. They can sense the signals in the circulating blood and send information to other brain regions to maintain homeostasis and regulate the autonomic nervous system. Recent studies showed that neural stem cells exist in these areas in addition to in the subventricular zone and subgranular zone in the dentate gyrus and that tanycyte-like cells in these organs express neural stem cell markers (Prevot et al., 2018; Furube et al., 2020). *Sulf1*-expressing cells seem to correspond to tanycytes, suggesting the possible role of *Sulf1* in neurogenesis. Furthermore, it is also intriguing that tanycytes in the adult brain and radial glial cells in the embryonic brain share some features and that tanycytes are thought to be genealogical descendants of radial glial cells because *Sulf1/2* are highly expressed in the radial glial cells in the third ventricle of mouse embryos and regulate Slit2 protein localization for accurate guidance of corticospinal tract axons (Okada et al., 2017).

Taken together, our findings suggest possible roles of *Sulf1* in dopaminergic transmission, NAc- and TS-associated behaviors, olfactory signaling, and maintenance of homeostasis. Future studies will be required to evaluate the relevance of *Sulf1* in these brain functions.

## Data Availability Statement

The raw data supporting the conclusions of this article will be made available by the authors, without undue reservation.

## Ethics Statement

The animal study was reviewed and approved by Animal Care and Use Committee of the University of Tsukuba.

## Author Contributions

KM, KK-M, TO, and MM designed the research and performed the experiments. KK generated the AAV virus. KM, KK-M, TO, and MM wrote the manuscript. All authors read and approved the final manuscript.

## Conflict of Interest

The authors declare that the research was conducted in the absence of any commercial or financial relationships that could be construed as a potential conflict of interest.

## Publisher’s Note

All claims expressed in this article are solely those of the authors and do not necessarily represent those of their affiliated organizations, or those of the publisher, the editors and the reviewers. Any product that may be evaluated in this article, or claim that may be made by its manufacturer, is not guaranteed or endorsed by the publisher.

## Footnotes

1
https://portal.brain-map.org/

## Supplementary Material

Supplementary Figure 1 Specificity of RNA probes in *in situ* hybridization. Coronal brain slices were hybridized with digoxigenin-labeled antisense RNA probes, and the signals were detected using a chromogenic substrate BM purple. *Sulf1* probes **(A–C)** and *Sulf2* probes **(D–F)** gave rise to distinct signals in the wild-type (WT) brain slices. The *Sulf1* and *Sulf2* signals were completely abolished in the *Sulf1* KO **(A’–C’)** and *Sulf2* KO **(D’–F’)** brain slices, respectively. The scale bar indicates 500 μm. Approximate AP levels from the bregma (in mm) are 2.0 **(A,A’,D,D’)**, 1.0 **(B,B’,E,E’)**, and –1.0 **(C,C’,F,F’)**.

Supplementary Figure 2 *Sulf* mRNA expression in the cerebral cortex. **(A)** *Sulf1* and *Sulf2* expressions in the prefrontal (PFC), primary motor (M1), primary somatosensory (S1), primary auditory (A1), and primary visual (V1) cortices are shown. The dotted lines in PFC indicate the midline; the slices above the lines show the contralateral side. The *Sulf1*, but not *Sulf2*, signals show the rostrocaudal gradient. **(B)** Layer-specific expression of *Sulf1* and *Sulf2* in the cerebral cortex. Microscopic images of the primary somatosensory cortex are shown. *Sulf1/2*-expressing cells were identified by staining with anti-β-galactosidase antibody (green). The cytoarchitecture was visualized using NeuroTrace Fluorescent Nissl Stain (red). The *Sulf1* signals were strong in layer 6b and weak in layer 6a, whereas the *Sulf2* signals were strong in layers 5 and 6b and weak in layer 6a. The scale bar indicates 100 μm. Approximate AP levels from the bregma (in mm) are 2.0 (PFC), 1.0 (M1), 0.5 (S1), –2.5 (A1), and –3.0 (V1).

Supplementary Figure 3 Experimental procedure for analyzing the co-expression of Sulf1 and the dopamine D1/D2 receptors. Representatives images of the brain slices show Sulf1 expression detected by β-galactosidase immunostaining and D2 expression detected by mCherry in the Sulf1^lacZ/+^;Drd2-Cre mouse that received AAV5-hSyn-DIO-mCherry injection in the nucleus accumbens. Confocal images of the coronal slice containing the nucleus accumbens are shown. An image analysis software, Imaris, was used to mark *Sulf1*-expressing cells, labeled green, with spheres in one layer, and D2-expressing cells, labeled red, with squares in another layer. After independent marking, the two images were merged and the doubly or singly marked cells were counted. The scale bar indicates 50 μm. The approximate AP level from the bregma (in mm) is 1.0.

## Abbreviations

A1: primary auditory area
AAV: adeno-associated virus
ac: anterior commissure
ACA: anterior cingulate area
AD: anterodorsal thalamic nucleus
AON: anterior olfactory nucleus
AP: anterior-posterior
ap: area postrema
Aq: aqueduct
ARH: arcuate hypothalamic nucleus
AVPV: anteroventral periventricular nucleus
BMA: basomedial amygdalar nucleus
BST: bed nucleus of the stria terminalis
cc: corpus callosum
CLA: claustrum
CM: central medial thalamic nucleus
CN: cerebellar nuclei
CP: choroid plexus
Cx: cerebral cortex
D1R: dopamine receptor type 1
D2R: dopamine receptor type 2
df: dorsal fornix
DIG: digoxigenin
DIO: double-floxed inverted open reading frame
DMH: dorsomedial nucleus of the hypothalamus
DP: dorsal peduncular cortex
DR: dorsal raphe nucleus
DSt: dorsal striatum
DV: dorsal-ventral
ECM: extracellular matrix
ECU: external cuneate nucleus
ENTl: entorhinal area lateral part
EPd: endopiriform nucleus dorsal part
f: fornix
fi: fimbria
fr: fasciculus retroflexus
GFP: green fluorescent protein
HPF: hippocampal formation
HS: heparan sulfate
HSPG: heparan sulfate proteoglycan
ILA: infralimbic area
IMD: intermediodorsal thalamic nucleus
IO: inferior olivary complex
IPN: interpeduncular nucleus
KO: knockout
LH: lateral hypothalamic area
LS: lateral septal nucleus
M1: primary motor area
M2: secondary motor area
MEPO: median preoptic nucleus
MH: medial habenula
ML: medial-lateral
mlf: medial longitudinal fasciculus
MnR: median raphe nucleus
MS: medial septal nucleus
MSN: medium spiny neuron
mt: mammillothalamic tract
NAc: nucleus accumbens
NAcSh: nucleus accumbens shell
NDB: diagonal band nucleus
NST: nucleus of the solitary tract
opt: optic tract
OT: olfactory tubercle
OVLT: organum vasculosum of the lamina terminalis
PAG: periaqueductal gray
PBS: phosphate-buffered saline
PBST: phosphate-buffered saline with 0.1% Tween-20
PCN: paracentral thalamic nucleus
PF: parafascicular thalamic nucleus
PFA: paraformaldehyde
PFC: prefrontal cortex
PG: pontine gray
Pir: piriform cortex
PL: prelimbic area
Pu: Purkinje cell layer
PVH: paraventricular hypothalamic nucleus
PVT: paraventricular thalamic nucleus
RE: reuniens thalamic nucleus
RN: raphe nuclei
ROI: region of interest
RSP: retrosplenial area
RT: reticular thalamic nucleus
S1: primary somatosensory area
SDS: sodium dodecyl sulfate
SFO: subfornical organ
SN: substantia nigra
SSC: saline-sodium citrate buffer
SVZ: subventricular zone
TBST: Tris-buffered saline with 0.1% Tween-20
TRN: tegmental reticular nucleus
TRS: triangular nucleus of septum
TS: the posterior tail of the striatum
TTd: tenia tecta dorsal part
TTv: tenia tecta ventral part
V1: primary visual area
V2: secondary visual area
vhc: ventral hippocampal commissure
VMH: ventromedial nucleus of the hypothalamus
VTA: ventral tegmental area
YFP: yellow fluorescent protein
ZI: zona incerta.
