# Supplementary figures and images for: Expression of Heparan Sulfate Endosulfatases in the Adult Mouse Brain: Co-expression of Sulf1 and Dopamine D1/D2 Receptors
Source: Front Neuroanat. 2021 Aug 20;15:726718. doi: 10.3389/fnana.2021.726718 (PMC8417564; doi:10.3389/fnana.2021.726718)

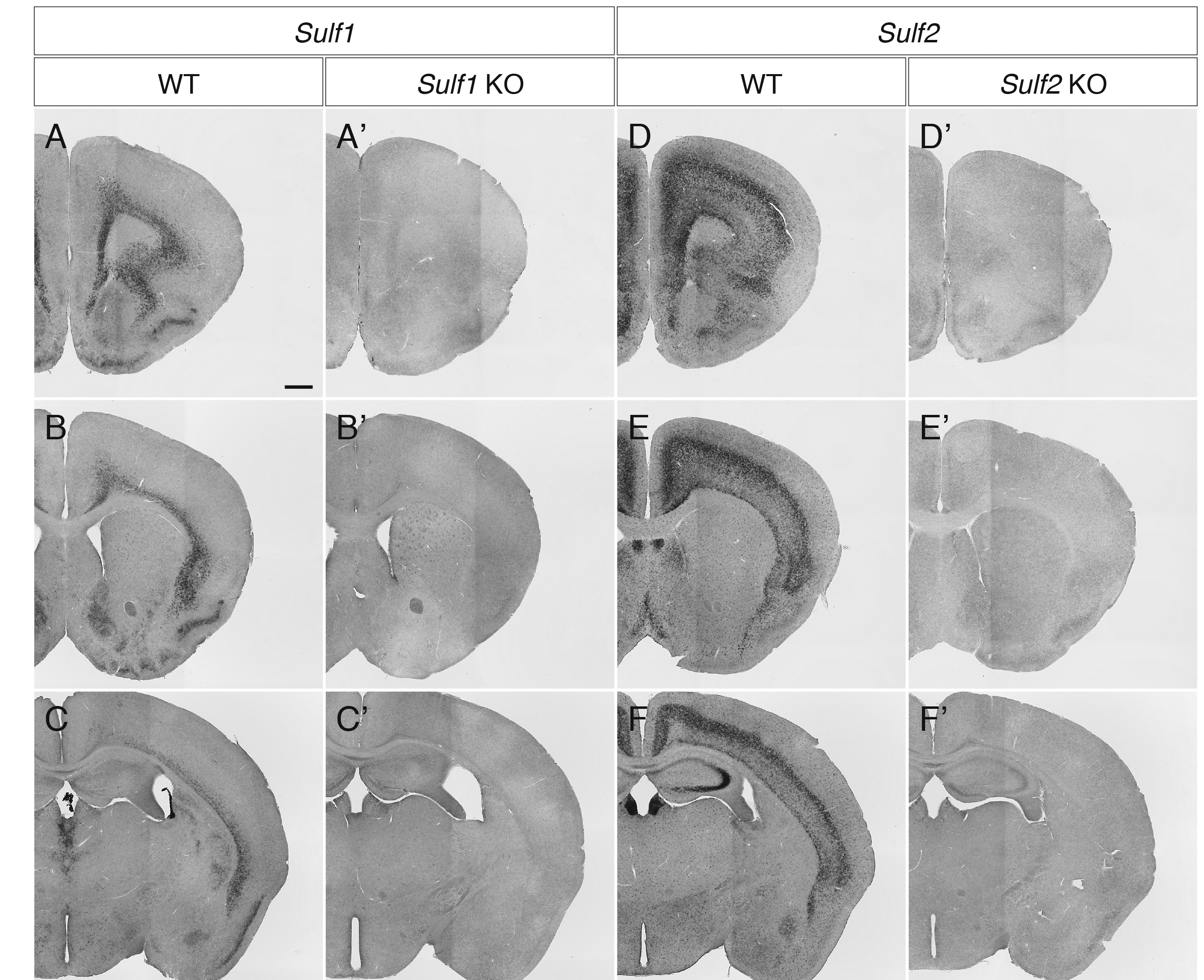

Supplement: Supplementary Figure 1 — Specificity of RNA probes in in situ hybridization. Coronal brain slices were hybridized with digoxigenin-labeled antisense RNA probes, and the signals were detected using a chromogenic substrate BM purple. Sulf1 probes (A–C) and Sulf2 probes (D–F) gave rise to distinct signals in the wild-type (WT) brain slices. The Sulf1 and Sulf2 signals were completely abolished in the Sulf1 KO (A’–C’) and Sulf2 KO (D’–F’) brain slices, respectively. The scale bar indicates 500 μm. Approximate AP levels from the bregma (in mm) are 2.0 (A,A’,D,D’), 1.0 (B,B’,E,E’), and –1.0 (C,C’,F,F’). [file Image_1.TIFF]

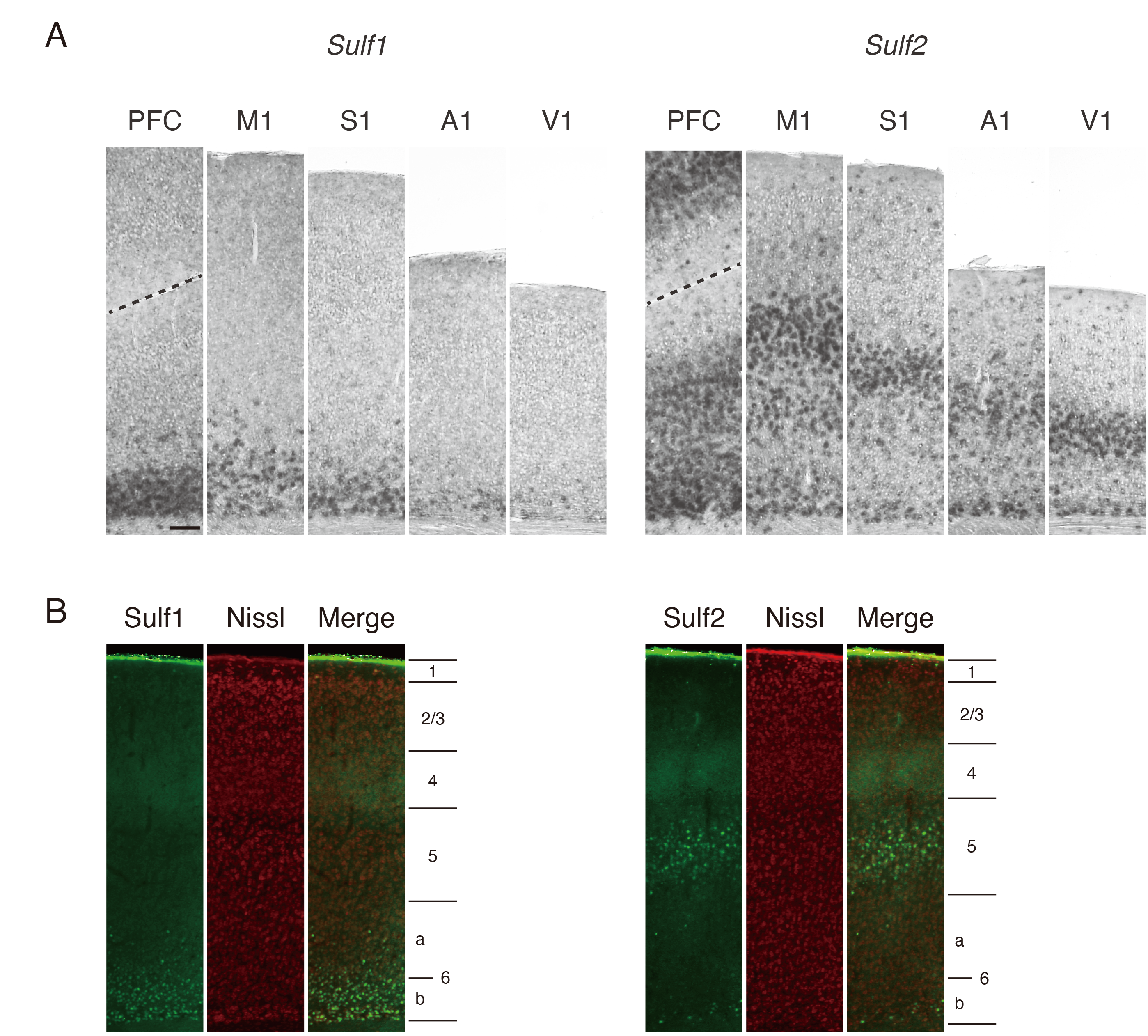

Supplement: Supplementary Figure 2 — Sulf mRNA expression in the cerebral cortex. (A) Sulf1 and Sulf2 expressions in the prefrontal (PFC), primary motor (M1), primary somatosensory (S1), primary auditory (A1), and primary visual (V1) cortices are shown. The dotted lines in PFC indicate the midline; the slices above the lines show the contralateral side. The Sulf1, but not Sulf2, signals show the rostrocaudal gradient. (B) Layer-specific expression of Sulf1 and Sulf2 in the cerebral cortex. Microscopic images of the primary somatosensory cortex are shown. Sulf1/2-expressing cells were identified by staining with anti-β-galactosidase antibody (green). The cytoarchitecture was visualized using NeuroTrace Fluorescent Nissl Stain (red). The Sulf1 signals were strong in layer 6b and weak in layer 6a, whereas the Sulf2 signals were strong in layers 5 and 6b and weak in layer 6a. The scale bar indicates 100 μm. Approximate AP levels from the bregma (in mm) are 2.0 (PFC), 1.0 (M1), 0.5 (S1), –2.5 (A1), and –3.0 (V1). [file Image_2.TIFF]

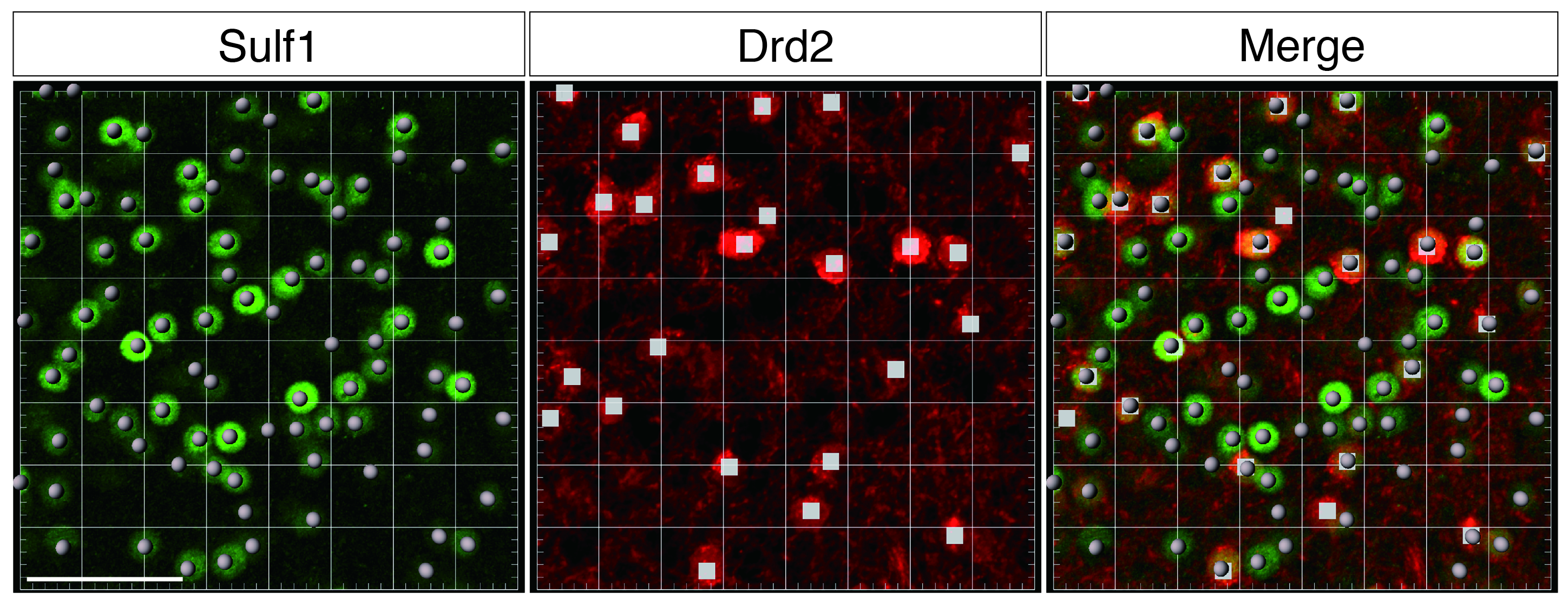

Supplement: Supplementary Figure 3 — Experimental procedure for analyzing the co-expression of Sulf1 and the dopamine D1/D2 receptors. Representatives images of the brain slices show Sulf1 expression detected by β-galactosidase immunostaining and D2 expression detected by mCherry in the Sulf1lacZ/+;Drd2-Cre mouse that received AAV5-hSyn-DIO-mCherry injection in the nucleus accumbens. Confocal images of the coronal slice containing the nucleus accumbens are shown. An image analysis software, Imaris, was used to mark Sulf1-expressing cells, labeled green, with spheres in one layer, and D2-expressing cells, labeled red, with squares in another layer. After independent marking, the two images were merged and the doubly or singly marked cells were counted. The scale bar indicates 50 μm. The approximate AP level from the bregma (in mm) is 1.0. [file Image_3.TIFF]
